# Early outbreak detection by linking health advice line calls to water distribution areas retrospectively demonstrated in a large waterborne outbreak of cryptosporidiosis in Sweden

**DOI:** 10.1186/s12889-017-4233-8

**Published:** 2017-04-18

**Authors:** Pär Bjelkmar, Anette Hansen, Caroline Schönning, Jakob Bergström, Margareta Löfdahl, Marianne Lebbad, Anders Wallensten, Görel Allestam, Stephan Stenmark, Johan Lindh

**Affiliations:** 1 Department of Monitoring and Evaluation, Public Health Agency of Sweden, 171 83 Solna, Sweden; 2 Department of Microbiology, Public Health Agency of Sweden, Solna, Sweden; 3 Department of Medical Sciences, Uppsala University, Uppsala, Sweden; 4 Department of Clinical Microbiology, Umeå University, Umeå, Sweden; 5 Department of Cell and Molecular Biology, Uppsala University, Uppsala, Sweden

**Keywords:** Early outbreak detection, Cryptosporidiosis, Syndromic surveillance, *Cryptosporidium hominis*

## Abstract

**Background:**

In the winter and spring of 2011 a large outbreak of cryptosporidiosis occurred in Skellefteå municipality, Sweden. This study summarizes the outbreak investigation in terms of outbreak size, duration, clinical characteristics, possible source(s) and the potential for earlier detection using calls to a health advice line.

**Methods:**

The investigation included two epidemiological questionnaires and microbial analysis of samples from patients, water and other environmental sources. In addition, a retrospective study based on phone calls to a health advice line was performed by comparing patterns of phone calls between different water distribution areas.

**Results:**

Our analyses showed that approximately 18,500 individuals were affected by a waterborne outbreak of cryptosporidiosis in Skellefteå in 2011. This makes it the second largest outbreak of cryptosporidiosis in Europe to date. *Cryptosporidium hominis* oocysts of subtype IbA10G2 were found in patient and sewage samples, but not in raw water or in drinking water, and the initial contamination source could not be determined. The outbreak went unnoticed to authorities for several months. The analysis of the calls to the health advice line provides strong indications early in the outbreak that it was linked to a particular water treatment plant.

**Conclusions:**

We conclude that an earlier detection of the outbreak by linking calls to a health advice line to water distribution areas could have limited the outbreak substantially.

**Electronic supplementary material:**

The online version of this article (doi:10.1186/s12889-017-4233-8) contains supplementary material, which is available to authorized users.

## Background

The protozoan parasite *Cryptosporidium* is a major cause of gastroenteritis in humans worldwide [1]. At least 29 valid species of *Cryptosporidium* have been identified [2] and the two most common species infecting humans are *Cryptosporidium parvum* and *Cryptosporidium hominis* [3]. *Cryptosporidium hominis* has been the cause of several large waterborne outbreaks. The largest took place in 1993 in Milwaukee, USA, where more than 400,000 people were infected [4]. Cryptosporidiosis is mainly transmitted by the fecal-oral route, usually through oocyst-contaminated water or food, or through contact with infected humans or animals. As few as 10 ingested oocysts can cause infection [5]. Asymptomatic carriage occurs [6, 7] while symptomatic infection is associated with diarrhoea, abdominal pain, nausea, vomiting and fever that usually resolve within 2 weeks. Symptoms occur a few days up to 2 weeks after ingestion of oocysts [5]. Severe life-threating diarrhoea may develop among immunocompromised patients [8]. Gastrointestinal- and joint symptoms can persist for several months after the initial infection with *Cryptosporidium* [9]. The public health impact of the parasite was recognised in Sweden in 2004 when cryptosporidiosis became a notifiable disease and the parasite was also included in the World Health Organization’s Neglected Diseases Initiative in the same year [10]*.*

In November and December 2010 a massive waterborne outbreak of *C. hominis* occurred in the city of Östersund in Jämtland County, Sweden. Based on a retrospective cohort study, it was concluded that approximately 27,000 individuals were infected through the drinking water which made it the second biggest reported waterborne outbreak of cryptosporidiosis globally [11]. A couple of months later, in April 2011, drinking water from a municipal water treatment plant (WTP) in the neighboring county of Västerbotten was suspected to be the source of a number of cases of cryptosporidiosis. A boil water notice (BWN) was therefore issued on 19 April and a web-based questionnaire was immediately created and published on the webpage of the municipality to collect epidemiological data. In order to complement the web-based questionnaire and better estimate the extent of the outbreak and to find its source, an additional study based on a postal questionnaire was performed in June 2011. The study was managed by the Västerbotten County Medical Office and the municipal environmental and health authorities in collaboration with the Public Health Agency of Sweden (at the time named Swedish Institute for Communicable Disease Control).

Syndromic surveillance is defined as the real-time (or near real-time) collection, analysis, interpretation and dissemination of health-related data [12]. As part of the ongoing effort of development and evaluation of a syndromic surveillance system at the Public Health Agency, a retrospective analysis of phone calls to a national health advice line from inhabitants living in Skellefteå municipality during the time period of the outbreak was performed. No contemporaneous analysis of the phone calls was performed at the time of the outbreak. A new approach for early detection and improved situational awareness of local waterborne outbreaks was used where call patterns from individuals living in different drinking-water distribution areas were compared. The utility of syndromic surveillance systems for detecting and tracking local gastrointestinal outbreaks (GI) has been questioned [13] but systems based on data from health advice lines have been shown to be successful in a few cases [14, 15]. Globally, there are several examples of similar syndromic surveillance systems based on health advice lines [16–18].

**Fig. 1:**
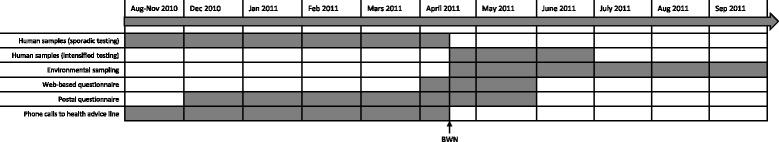
Time line indicating for which time periods the different data sources were used in the analyses

This study describes the outbreak investigation by summarizing the results from web-based and postal questionnaires, human and environmental sampling and the analysis of phone calls to the health advice line. It outlines the extent and duration of the outbreak, risk factors and clinical characteristics of the infected persons, and discusses the potential for detecting the outbreak earlier. Figure 1 depicts a time line indicating for which time periods the different data sources were used in the analyses.

## Methods

### Study setting

Skellefteå is situated in Västerbotten County geographically located next to Jämtland County where the Östersund outbreak occurred. The distance between Skellefteå and Östersund is almost 500 km. Skellefteå is a municipality with a population of approximately 72,000 inhabitants. Twenty-eight water treatment plants are operating within the municipality. Two of these deliver water to the city of Skellefteå; Slind WTP and Abborrverket WTP, where the latter delivers water to the majority of the inhabitants. All water treatment plants in the municipality use groundwater as the water source except Abborrverket which uses surface water obtained from the river Skellefteälven. Abborrverket WTP produces approximately 18,000 m^3^ of treated water daily to 44,000 of the 72,000 inhabitants in the municipality (31 March 2011). The normal water intake to Abborrverket is located far out and deep in the river but due to icing during the winter months the intake is shifted to a more shallow position closer to shore where the ice can be removed more easily.

### Microbiological investigation

#### Human samples

Fecal samples from patients seeking healthcare for gastrointestinal illness were analysed with standard techniques for enteric bacterial pathogens; polymerase chain reaction (PCR) for analysis of noro- and sapoviruses and microscopy for analysis of *Entamoeba* spp. and *Giardia intestinalis.* Samples were only sporadically analysed for presence of *Cryptosporidium* oocysts up until 19 April 2011, when the current outbreak was first suspected. An intensification of testing for *Cryptosporidium* followed from that time until 1 July 2011 when the outbreak was considered over. Samples tested for *Cryptosporidium* were analysed using standard concentration technique followed by modified Ziehl-Neelsen staining [19]. A subset of positive *Cryptosporidium* samples (*n* = 26) were sent to the Swedish Institute for Communicable Disease Control for species identification by PCR restriction fragment length polymorphism (RFLP) analysis of the rRNA gene [20, 21]. Subtypes were characterized by sequence analysis of the 60 kDa glycoprotein (gp60) gene [22, 23].

#### Environmental samples

At the time of the outbreak Abborrverket WTP used flocculation and sedimentation followed by sand filtering and chlorination for water treatment. This water treatment setup could be sufficient for removal of *Cryptosporidium* oocysts if the processes work optimally and the concentration of oocysts is relatively low, but ultraviolet (UV) treatment is generally preferred as a disinfectant [24]. The winter intake was used from 19 November 2010 until 19 April 2011. A total of 38 samples were collected from the drinking water system during a period of 5 months, 19 April to 15 September 2011. These samples included raw water from the river Skellefteälven, i.e. incoming water to Abborrverket WTP, treated water at Abborrverket WTP and samples taken from the distribution net.

Twelve influent and twelve effluent wastewater samples were collected at the main sewage water treatment plant (SWTP) Tuvan. Moreover, in order to investigate possible causes of contamination of Skellefteälven and to trace sources of oocysts, 9 samples were collected from the wastewater and storm water systems and from other relevant locations.

Water samples were analysed for *Cryptosporidium* oocysts according to ISO 15553:2006 [25] with filtration of water (10–1000 L), immunomagnetic separation (IMS) and immunofluorescence (IFL) microscopy. The slides with concentrated and purified material were identified by fluorescent-marked oocysts specific in size, shape, internal structure and DAPI-(4′,6-diamidino-2-phenylindole)-stained nuclei. Wastewater samples were analyzed as water samples but without passing filters and in smaller volumes, 50–100 mL for influent wastewater and 0.3–0.5 L for effluent wastewater. Two sediment samples from the inside of the influent raw water pipe were also analysed as water sample but without filtration before IMS. DNA from one wastewater concentrate was analysed by sequence analysis of the gp60 gene as described for human samples [22, 23].

### Epidemiological investigation

#### Web-based questionnaire

The same day as the BWN was issued, on 19 April 2011, a web-based questionnaire (Additional file 1) was created in order to immediately start collecting epidemiological data. The value of such a questionnaire was demonstrated in the preceding cryptosporidiosis outbreak in Östersund [11] and those experiences were applied here as well. The questionnaire was made available to the public on the website of the municipality on the evening the same day, and was closed on 9 May 2011. The public was informed of the questionnaire by press releases and there were also links to it from key web pages such as the local newspaper and Västerbotten County Council. The full data set was summarised after the outbreak was considered to be over. Visitors to the webpage who were residents of Skellefteå municipality, both individuals with and without GI symptoms, were asked to answer a set of questions regarding gastrointestinal illness in the family. A case attributed to the outbreak was defined as a person with residential address within Skellefteå municipality with ≥3 loose stools per day for at least 1 day with onset between 1 April and 5 May 2011. Respondents with a date of symptom onset before 1 April or after 5 May, persons who had travelled abroad 2 weeks prior to symptom onset, as well as individuals with a residential postal code outside Västerbotten County were excluded from the analysis. Remaining respondents who did not fulfil the criteria of having ≥3 loose stools per day were considered non-cases. More detailed analyses of the data were not performed since the follow-up postal survey was conducted.

#### Postal questionnaire

A retrospective cohort study was performed in June 2011 by sending a questionnaire to a random sample of 1754 citizens in the municipality of Skellefteå (Additional file 2, Additional file 3). The random sample was stratified by age (0–5 years, 6–15 years, 16–65 years and 66 years or older) and gender. Questions were asked to find out about the start and magnitude of the outbreak, the source of the outbreak and risk factors for disease. The questionnaire contained questions on demographics, onset, duration and occurrence of symptoms indicating cryptosporidiosis, and water consumption as well as history of symptoms before 1 January 2011. Caretakers were asked to answer for children <15 years of age. A case attributed to the outbreak was defined as a person with ≥3 loose stools per day for at least 1 day with onset between 1 December 2010 and 31 May 2011.

#### Statistical analysis of the postal questionnaire

Each of the 1754 respondents were assigned a random number and a barcode on the questionnaire was used to identify each respondent. The postal codes were matched to the water distribution areas of the WTPs. In a stratified survey study, weights are used to calculate the number of individuals in the population represented by each individual in the sample. Binary logistic regression was used to find associated variables for the propensity of responding to the survey. Age, gender and water supply were used to calibrate the weights for non-response to adjust for unbalance between the sample and the population.

The association between the binary outcome of case/non-case and the exposure variables was analysed by binary logistic regression. Included in the model as covariates and exposure variables were gender, age (0–5 years, 6–15 years, 16–65 years, and 66 years or older), gastric ulcer (yes, no), irritable bowel syndrome (yes, no), Crohn’s disease (yes, no), celiac disease (yes, no), lactose intolerance (yes, no), immunodeficiency disease (yes, no), average tap water consumption (<1 glass, 1 glass, 2–5 glasses, >5 glasses) and household water supply (Abborrverket, not Abborrverket or not from any WTP/own well).

The results from the binary logistic regression were expressed as odds ratios (OR). All “I do not know” answers for binary questions were regarded as non-informative and were set as missing values prior to the analysis. Missing values for binary variables were then given a value (yes, no) using multiple imputation chain equations [26]. The chains contained all exposure variables plus the outcome non-case/case [27]. Twenty datasets with different imputed values for missing data were created and binary logistic regression results from each dataset were weighted together into one result using Rubin’s formula [28]. All analyses were performed in the statistical software R (version 3.3.2) using the packages survey (version 3.31.2), MICE (version 2.25) and the generalized linear model function (glm) in the base R package stats. In all analyses a *p*-value less than 0.05 was used as a significant result and in case of estimated confidence intervals a confidence level of 95% was applied.

#### Analysis of phone calls to a health advice line

Healthcare Guide 1177 is a national Swedish telephone health advice line staffed by nurses. The service provides advice and information about urgent, but non-life-threatening, health problems. The medical record created for each consultation includes a structured data field, called the contact cause, that represents the most severe symptom as assessed by the nurse [29]. There are almost 200 contact causes in the service’s medical decision support system but only a handful are related to GI problems. For the purpose of this study daily call counts on GI symptoms were retrospectively extracted from the service for inhabitants in Skellefteå municipality from 1 August 2010 to 18 April 2011. The contact causes “vomiting or nausea”, “diarrhoea” and “stomach pain” were used since changes in contact patterns for these symptoms previously have been shown in outbreaks of cryptosporidiosis [14]. In addition, for each call, information on the postal code of the registered residence address of the patient was extracted.

Postal codes were divided into two geographical regions; belonging to the distribution area of Abborrverket WPT or not, and the number of inhabitants in the corresponding regions were calculated. To compare the call patterns of GI-related symptoms between these two regions a previously published outbreak detection algorithm [14] was used but with a minor modification. No analyses were performed for the period from the BWN and onwards since, as the information of an ongoing outbreak becomes public, the contact pattern to the health advice line changes drastically and it is challenging to adjust for this in the analyses.

The daily call count, *C*_*t* , *i*_, for one contact cause or a single group of contact causes at day *t* for geographical region *i* was classified as an outbreak signal if it exceeded a threshold *T*_*t* , *i*_:

$$ {T}_{t, i}= \max \left( L, V\right), $$

$$ V=\left({E}_{t, i}+ L\times {SD}_{t, i}\right), $$

$$ L\in \left\{3,5\right\} $$

$$ {E}_{t, i}={p}_{t, i}\times {N}_i, $$

$$ {SD}_{t, i}=\sqrt{N_i\times {p}_{t, i}\times \left(1-{p}_{t, i}\right)}, $$

$$ {p}_{t, i}=\frac{\sum_{j=1, j\ne i}^{n_i}{\sum}_{\tau}{\omega}_{\tau}{C}_{\tau, j}}{10\times \sum_{j=1, j\ne i}^{n_i}{N}_j}, $$

$$ \tau \in \left\{ t-7, t-8, t-9, t-10, t-11, t-12, t-13, t-14\right\}, $$

$$ {\omega}_{\tau}=\left\{\begin{array}{l}2, if\ \tau \in \left\{ t-7, t-14\right\}\\ {}1, if\ \tau \in \left\{ t-8, t-9, t-10, t-11, t-12, t-13\right\}\end{array}\right. $$

where *L* is the threshold level for a weak and strong outbreak signal respectively, *V* is the threshold for a positive outbreak signal, *E*_*t* , *i*_ is the expected number of calls and *SD*_*t* , *i*_ is the standard deviation, both based on a binomial distribution, *N*_*i*_is the population size of geographical region *i*, *p*_*t* , *i*_ is the probability of a single call per inhabitant per day, and *n*_*i*_ is the number of geographical regions in the analysis. In the current study, two geographical regions were included: Abborrverket distribution area and not Abborrverket distribution area. It is important to note that calls from inhabitants of the geographical region under investigation are not included in the calculation of its threshold since that would increase the threshold if an outbreak in that region has been ongoing for more than 6 days. Compared to the previously published algorithm, the time period for the calculation of *p*_*t* , *i*_ has been modified. Here, calls for 8 days, *t-7* to *t-14*, were included. Since the call patterns differ between different weekdays, call counts for the weekdays matching the day for which the threshold is calculated, *t-7* and *t-14*, has a weight of 2. The motivation for this modification of the algorithm was primarily to reduce the risk of calculating *p*_*t* , *i*_ based on small number of calls.

## Results

### Microbiological investigation

#### Human samples

Between 1 January and 1 July 2011, 145 laboratory confirmed cases of domestic cryptosporidiosis were reported from Västerbotten County. Only a handful were reported before 19 April, including one case on 15 April and two on 18 April. Genotyping identified *C. hominis* subtype IbA10G2 in samples from 24 confirmed cases, while no amplification product was obtained from the remaining two samples that were tested. No other gastrointestinal pathogens were found in a subset of the samples that were positive for *Cryptosporidium*.

#### Environmental samples

*Cryptosporidium* oocysts could not be detected in any of the 38 samples collected from the drinking water system. In influent and effluent wastewater samples from Tuvan SWTP oocysts were detected in 10 out of 24 samples. The concentration of oocysts in influent wastewater was highest on 22 April 2011 at 150,000 oocysts/L and declined to 6200 oocysts/L on 1 June 2011. From 27 June 2011 no oocysts were detected in influent wastewater. In effluent wastewater the concentration was highest on 8 June 2011 with a concentration of 12,000 oocysts/L. In subsequent samples the concentration varied between 1700 and 4200 oocysts/L and from 27 June 2011 the concentration was below the detection limit. Molecular investigation of one wastewater sample revealed *C. hominis* subtype IbA10G2. In the remaining 9 water and sediment samples collected at other places no oocysts were detected.

### Epidemiological investigation

#### Web-based questionnaire

**Fig. 2:**
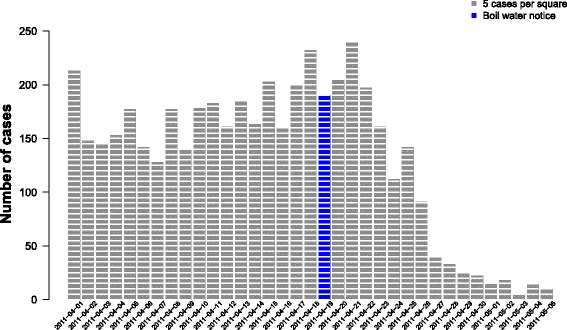
Epidemiological curve based on observed cases in the web questionnaire

The epidemiological curve based on the web-based questionnaire (Fig. 2) showed that the number of cases declined after a couple of days following the BWN and verified the hypothesis of an ongoing waterborne outbreak. Importantly, it also indicated that the outbreak started well before 1 April. In total 12,358 individuals answered the questionnaire and 11,065 remained after exclusions. The results from the questionnaire were continually monitored in order to provide information for decision making based on the extent of the outbreak, who were being affected and to follow up the effect of interventions. Moreover, it was used to inform the inhabitants about the progress of the outbreak and these reports were highly appreciated.

#### Postal questionnaire

**Fig. 3:**
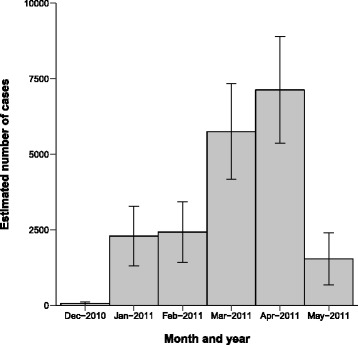
Epidemiological curve of population estimates of number of cases from the postal questionnaire

In total, 1099 out of 1754 (63%) questionnaires were answered and returned for analysis. The survey showed that 26.4% of the respondents fulfilled the case definition, i.e. self-reported diarrhoea (≥3 loose stools per day) between 1 December 2010 and 31 May 2011, which corresponds to an estimate of 18,449 cases (Table 1). The data from the survey also provided evidence that the outbreak started in January and ended by the end of May (Fig. 3). April was the peak month with 6969 cases. If the outbreak had been detected earlier and we assume that all cases from 1 February forward had remained healthy, the estimation is that the outbreak would have affected 2273 individuals, corresponding to approximately 12% the current outbreak size.

**Table 1:** Population estimates of cases and non-cases of *Cryptosporidium*-infection in Skellefteå between December 2010 and May 2011

Status	N	SE (N)	95% CI (N)	N (%)
Non-cases	51,618	1214	49,239 – 53,997	73.7
Cases	18,448	1191	16,114 – 20,782	26.3

*N* Number, *SE* standard error, *CI* confidence interval

Based on postal questionnaire

Only the variables age and water supply were identified as risk factors for infection (Table 2). Divided into age groups, children up to 5 years were most affected, 37.2%, while the group of 66 years and above were least affected, 12.1% (Table 3). Among the different water supplies in Skellefteå, water from Abborrverket WPT was the only supply that significantly correlated with an increased risk of infection (*p* < 0.001). Approximately 1 in 3 (32.7%) living in the distribution area of Abborrverket WPT had symptoms of cryptosporidiosis, compared to 16.2% of inhabitants living in other areas (Table 4). The most common symptoms, each present in more than 70% of the respondents that fulfilled the case definition, were fatigue, abdominal pain, upset stomach, and watery diarrhoea (Table 5).

**Table 2:** Significant risk factors for infection based on postal questionnaire

	Odds ratio	95% CI	*P*-value*
Age			
0-5	4.22	2.66 – 6.68	<0.001
6-15	2.28	1.42 – 3.68	0.001
16-65	3.08	1.96 – 4.83	<0.001
66-	1.00		
Water source			
Not from any WTP/own well	1.00		
Abborrverket WTP	2.30	1.49 – 3.56	<0.001
Not Abborrverket WTP	1.14	0.68 – 1.91	0.613

*CI* confidence interval, *WTP* water treatment plant

*Fisher’s exact *P* - value

**Table 3:** Population estimates of cases and non-cases of *Cryptosporidium*-infection in Skellefteå between December 2010 and May 2011 divided into age groups

Status	Age	N	SE (N)	95% CI (N)	N (%)
Non-cases	0-5	2433	121	2195 – 2671	62.8
	6-15	5542	208	5135 – 5950	75.3
	16-65	30,728	1148	28,479 – 32,977	69.6
	66-	12,914	326	12,276 – 13,553	87.9
Cases	0-5	1442	120	1207 – 1676	37.2
	6-15	1819	202	1423 – 2215	24.7
	16-65	13,402	1132	11,183 – 15,621	30.4
	66-	1786	301	1196 – 2377	12.1

*N* number, *SE* standard error; *CI* confidence interval

Based on postal questionnaire

**Table 4:** Population estimates of cases and non-cases of *Cryptosporidium*-infection in Skellefteå between December 2010 and May 2011 divided into water supply categories

Status	Category	N	SE (N)	95% CI (N)	N (%)
Non-cases	Not Abborrverket WTP	22,649	642	21,392 – 23,906	83.8
	Abborrverket WTP	28,696	1033	26,944 – 30,994	67.3
Cases	Not Abborrverket WTP	4368	624	3145 – 5590	16.2
	Abborrverket WTP	14,081	1019	12,084 – 16,078	32.7

*N* number, *SE* standard error, *CI* confidence interval

Based on postal questionnaire

**Table 5:** Clinical features of *Cryptosporidium*-infection cases in the municipality of Skellefteå during December 2010 to May 2011

	Proportion with symptom (%)	95% CI (%)
Fatigue	78.5	72.4 – 84.7
Abdominal pain	73.3	66.8 – 79.8
Upset stomach	71.4	64.7 – 78.2
Diarrhoea		
Watery	70.2	63.4 – 77.0
Bloody	0.9	0.0 – 2.5
Nausea	63.5	56.1 – 70.9
Headache	46.9	39.3 – 54.5
Vomiting	35.8	28.6 – 43.1
Fever >38 °C	36.5	28.2 – 42.8
Joint pain	27.4	20.4 – 34.4
Pain in eyes	14.6	9.1 – 20.1

*CI* confidence interval

Based on postal questionnaire

#### Health advice line

**Fig. 4:**
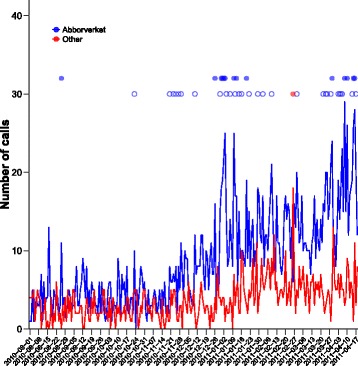
Daily call counts from Skellefteå municipality to Healthcare Guide 1177 regarding GI symptoms from 1 August 2010 until the day before the BWN on 19 April 2011. Inhabitants are divided into two groups; those living in the water distribution area of Abborrverket WPT (*blue*) and those who are not (*red*). Outbreak signals from the detection algorithm [14] are shown as *blue* (Abborrverket WTP) and red circles (not Abborrverket WTP) with weak (*hollow circles*) and strong outbreak signals (*filled circles*) along the lower horizontal and upper horizontal, respectively

Starting on 30 December 2010, the retrospective analysis showed a sequence of 6 days of consecutive outbreak signals regarding GI symptoms from individuals living in the distribution area of Abborrverket WTP (Fig. 4). Four of those were strong. A large number of outbreak signals for inhabitants living in the distribution area of Abborrverket WTP were evident during the following time period up until the BWN.

In contrast, very few outbreak signals regarding GI symptoms were present for Abborrverket WPT distribution area during the autumn and early winter of 2010. However, there was a small cluster of five outbreak signals between 20 November and 29 November 2010, but they were weak and not on consecutive days, hence the interpretation was that those outbreak signals were inconclusive. For the other geographical area in the analysis, individuals not living in the distribution area of Abborrverket WTP, only one weak outbreak signal was present during the entire time-period under investigation - emphasizing the abnormality of the identified outbreak signal pattern for the distribution area of Abborrverket WTP in the beginning of 2011. Similar results are obtained using the original definition of the outbreak algorithm and with other groupings of ages and contact causes related to cryptosporidiosis (results not shown).

When comparing the current outbreak signals based on geographical regions corresponding to the distribution of drinking water with outbreak signals based on (adult) GI calls from the entire Skellefteå municipality with respect to the other municipalities within Västerbotten County [14], the two patterns are similar and suggest the same time for outbreak detection: beginning of January 2011.

## Discussion

The three cases of cryptosporidiosis in the middle of April together with other indications from informal sources regarding large numbers of sick people, and higher-than-normal contacts regarding symptoms of gastrointestinal illness reported by the nurses staffing the regional health advice line, led the authorities to suspect an outbreak. However, our epidemiological investigation shows that the outbreak had already started, unnoticed to the authorities, in the beginning of January 2011. High norovirus activity together with only a handful of domestic cases of cryptosporidiosis reported in Västerbotten County between January and 19 April 2011 were contributing factors to the late detection of the outbreak. New routines are now in place in Västerbotten County where analysis of *Cryptosporidium* is performed on fecal samples if there are clusters of cases with gastrointestinal symptoms or other indications of an outbreak.

If the outbreak had been detected in the beginning of the year by systematic monitoring of the telephone calls as described in this study, it is very likely that the outbreak would have ended during January. Two facts support this conclusion. First, once the outbreak was suspected the BWN was an effective intervention that substantially limited illness within a few days. A similar delay in the decrease of reported cases was seen in the Östersund outbreak [11] and it is explained by the time it takes to develop symptoms after ingestion of oocyst. Second, the outbreak signals from the described analyses of telephone calls would have given a strong indication during January that the outbreak was waterborne and which drinking water supply to suspect (Abborrverket WTP). We therefor argue that such an early outbreak detection followed by a timelier BWN in January 2011 would have limited the outbreak substantially – from approximately 18,500 cases down to 2300 cases if all who fell ill after 31 January had remained healthy.

The potential of syndromic surveillance systems based on analysing telephone call patterns to Healthcare Guide 1177 for early event detection and situational awareness of local outbreaks has been shown previously [14]. In the current work the concept was taken a step further by comparing call patterns between water distribution areas that were based on groups of postal codes. The importance of this should not be underestimated. In the situation of an unknown waterborne outbreak, or other types of local outbreaks where the spread matches geographical areas used in the analysis, this procedure gives a more timely indication of the underlying cause and therefore substantially increases the chances of effective countermeasures. Since water distribution areas are known, the approach can be used in systems for syndromic surveillance.

There is always a tradeoff between sensitivity and specificity in signal detection. In practical terms it is the institution that is eligible to act on the signal that needs to find a reasonable protocol for signal evaluation and validation. To increase the sensitivity and hence the potential of timely detection of local outbreaks, which usually are very short-lived in contrast to the outbreak under investigation here, the outbreak algorithm used operates on a daily basis. This has the drawback of reduced specificity, i.e. that more false positive outbreak signals are generated due to randomness, especially for geographical regions where the population size is small. Despite this, and even though the daily call counts are relatively low, the outbreak signal pattern shown in Fig. 4 is exceptional and clearly indicates that individuals living in the distribution area of Abborrverket WTP report more GI symptoms compared to individuals living in other areas. Moreover, this outbreak signal pattern is similar if other groupings of age and contact causes related to symptoms of cryptosporidiosis are used.

Although possible sources of contamination were investigated and discussed no conclusive information could be found. Several samples from the drinking water system and the environment were analysed for *Cryptosporidium* oocysts but none were detected - apart from the findings in wastewater samples. The most likely theory in our opinion is that the winter intake of water to Abborrverket WTP, which is more exposed to contamination since it is located more shallowly in the river and closer to shore compared to the summer intake, was contaminated with *Cryptosporidium* oocysts from sewage from one or several sources. However, data on weekly maximal turbidity and bacteriological counts (*Escherichia coli*, general coliform bacteria, enterococci and *Clostridium perfringens*) in raw water to Abborrverket WTP for the period October 2010 to March 2011 had been within normal levels so such a contamination, if present, was not detected in the routine testing at the WTP.

The water intake was shifted to the summer position on the same day as the BWN was issued on 19 April 2011, which may explain why no *Cryptosporidium* oocysts were found in the water samples, since they were all taken after 19 April. It is worth noting that the outbreak signals from the syndromic surveillance algorithm present in November 2010 coincide with the shift to the winter intake which might indicate a contamination at that point in time as well - although probably unrelated to the current outbreak. The fact that *Cryptosporidium* oocysts were found in wastewater is in our opinion related to the fact that a substantial part of the population connected to the municipal SWTP Tuvan was infected with *C. hominis* IbA10G2. Thus, although the initial cause of the outbreak remains unknown, it was most certainly caused by fecal contamination of human origin since *C. hominis* is almost exclusively human specific.

Compared to the Östersund outbreak [11] only age was the common risk factor. In contrast, the current study did not find statistical significance for any of the underlying diseases nor amount of water consumed. Symptom profiles were almost identical between the outbreaks. The same *Cryptosporidium* gp60 subtype was found responsible for both the current and the Östersund outbreak. It is in our opinion likely that the outbreaks were related since this subtype seldom is found in domestic cases in Sweden, in contrast to other European countries, and the time period between the outbreaks was short. However, similarity on gp60 is not conclusive evidence [30] and the question of whether two outbreaks were related is currently investigated by whole genome sequencing. Speculatively, one or a few infected individuals from the Östersund outbreak brought the parasite to Skellefteå and caused a second outbreak through spread of *Cryptosporidium* oocysts via sewage, into the river, finally ending up in the drinking water since the microbial barriers present in Abborrverket WPT at the time were insufficient to inactivate or remove the oocysts.

After the outbreak was identified, the water distribution system was flushed to remove the contamination and work to improve the water treatment in Abborrverket WTP was started. Since the large outbreak in Östersund only took place a few months earlier the municipality of Skellefteå could utilize experience from the actions taken to stop the former outbreak. Even so, the BWN had to be kept in place for 5 months, compared to 3 months in Östersund. This was partly due to the longer period of time it took to install an ultraviolet unit as an additional microbiological barrier in Abborrverket WTP as well as a longer and more complex water distribution network that had to be flushed. As of November 2016, the municipality of Skellefteå is in the process of rebuilding their infrastructure for production of drinking water. This work had started before the outbreak of cryptosporidiosis.

## Conclusions

Our investigation concludes that approximately 18,500 people in the municipality of Skellefteå were infected by *Cryptosporidium* during the winter and spring of 2011 making it the second largest outbreak of cryptosporidiosis described in Europe to date. *Cryptosporidium hominis* subtype IbA10G2 was isolated from patient samples and wastewater. The epidemiological investigation strongly indicates that this outbreak was waterborne based on the vast number of cases, as well as the fact that the BWN were an effective countermeasure, and that people living in the water distribution area of one specific WTP were more likely to become ill. This conclusion is also strongly supported by the pattern of phone calls to the national health advice line Healthcare Guide 1177. We therefore firmly believe that the outbreak was waterborne and caused by *C. hominis* transmitted through the public water supplied by Abborrverket WTP even though no oocysts could be found in raw water or in drinking water.

Moreover, our results show that the outbreak went unnoticed to the authorities for several months and that systematic monitoring of phone calls to the health advice line, as described in this study, could have limited the outbreak to approximately 2300 cases compared to the current estimate of 18,500 cases. This new approach of linking health advice line calls to water distribution areas has been implemented in a system for syndromic surveillance deployed by the Public Health Agency of Sweden in 2016.

## Additional files

Additional file 1: Web-based questionnaire. Translated web-based questionnaire. (PDF 162 kb)

Additional file 2: Postal questionnaire for adults. Translated postal questionnaire for adults. (PDF 128 kb)

Additional file 3: Postal questionnaire for children. Translated postal questionnaire for children. (PDF 128 kb)

## Abbreviations

BWN: Boil water notice
GI: Gastrointestinal
IFL: Immunofluorescence
IMS: Immunomagnetic separation
OR: Odds ratio
PCR: Polymerase chain reaction
RFLP: Fragment length polymorphism
SWTP: Sewage water treatment plan
UV: Ultraviolet
WTP: Water treatment plant
