# Supplementary material for: Early outbreak detection by linking health advice line calls to water distribution areas retrospectively demonstrated in a large waterborne outbreak of cryptosporidiosis in Sweden
Source: BMC Public Health. 2017 Apr 18;17:328. doi: 10.1186/s12889-017-4233-8 (PMC5395832; doi:10.1186/s12889-017-4233-8)
Supplement: Supplementary file 1 — Web-based questionnaire. Translated web-based questionnaire. (PDF 162 kb) [file 12889_2017_4233_MOESM1_ESM.pdf]

We encourage all inhabitants in Skellefteå municipality to answer this questionnaire even though you have not been ill. The purpose of the questionnaire is to estimate the number of people in Skellefteå who has been/is ill in gastrointestinal disease since April 1. Your participation is important in order to try to understand the cause of the outbreak. The questionnaire is part of the ongoing outbreak investigation, which is performed together with Skellefteå municipality, the Västerbotten County Medical Office and Swedish Institute for Communicable Disease Control. If both you and your child/children has been ill, fill in a separate questionnaire for each person. The questionnaire will take approximately 5 minutes to fill in and the complete questionnaire should be completed at the same time. All answers will be handled confidentially and analyses will be performed with anonymized data. Thank you for your participation!

Questions

Gender:	Male Female
Year of birth:	YYYY
Address at home	Postal code, Place, Address
Address at work/school/pre-school	Postal code, Place
Have you been ill in gastrointestinal disease after 1 April 2011?	Yes No Do not know
When did you get ill in gastrointestinal disease (date of symptom onset)?	YYYY-MM-DD

Which of the following symptoms do you have/did you have during your gastrointestinal disease?

- Diarrhoea (3 or more loose stools per day)	Yes No Do not know
- Bloody diarrhea	Yes No Do not know
- Stomach pain	Yes No Do not know
- Upset stomach (e.g. gases)	Yes No Do not know
- Vomiting	Yes No Do not know
- Nausea	Yes No Do not know
- Fever, above 38 degrees	Yes No Do not know
- Other symptoms	Yes No Do not know

If you have had other symptoms, please state those:

Have you been admitted to hospital for you gastrointestinal illness?	Yes No
- If so, for how many days?	Number of days
Have you travelled abroad during the 14 days before symptom onset (including travels to the nordic countries)?	Yes No Do not know

- If so, where did you travel and between which dates

Have you eaten at any of the following sites since 15 Mars?

- Restaurant?	Yes No Do not know
o If so, please state which one/ones.	
- Café or confectionery?	Yes No Do not know
o If so, please state which one/ones.	
- Snack bar?	Yes No Do not know
o If so, please state which one/ones.	
- School canteen?	Yes No Do not know

- If so, please state which one/ones.

- Personnel canteen? Yes No Do not know
  - If so, please state which one/ones.
- Other/take away? Yes No Do not know
  - If so, please state which one/ones.

What type of household water supply do you have?

Municipal water

Own well

Other joint facility

Do not know

- If you have any other household water supply, please state it:

How many glasses of water do you drink on average per day?

< 1 glass

1 glass

2-5 glasses

>5 glasses

Do not know

Have you consumed water from a stream or a lake since 15 Mars? Yes No Do not know

Have you bathed in a public swimming pool since 15 Mars? Yes No Do not know

- If so, which public swimming pool?

Have someone else in your household been ill in gastrointestinal disease during the last month? Yes No Do not know

- Don't forget to fill in a questionnaire for that person in such a case.

Do you have any idea of what you became ill?

If you have other comments about your illness, please state those:
