# Supplementary material for: Early outbreak detection by linking health advice line calls to water distribution areas retrospectively demonstrated in a large waterborne outbreak of cryptosporidiosis in Sweden
Source: BMC Public Health. 2017 Apr 18;17:328. doi: 10.1186/s12889-017-4233-8 (PMC5395832; doi:10.1186/s12889-017-4233-8)
Supplement: Supplementary file 2 — Postal questionnaire for adults. Translated postal questionnaire for adults. (PDF 128 kb) [file 12889_2017_4233_MOESM2_ESM.pdf]

Questionnaire gastrointestinal disease/cryptosporidium adult

Section 1: Disease information regarding the outbreak of Cryptosporidium

Which date do you respond to this questionnnaire? Month: Day:

1a. Have you, since the first of January 2011 had stomach problems? Yes No Do not know

If you did not have any stomach problems, continue with question 8.

1b. Which type of stomach problems have you had since first of January?

- diarrhoea with 3 or more loose stools per day	Yes	No	Do not know
- watery diarrhoea	Yes	No	Do not know
- bloody diarrhea	Yes	No	Do not know
- abdominal pain/stomach cramps	Yes	No	Do not know
- upset stomach (e.g. flatulence)	Yes	No	Do not know
- vomiting	Yes	No	Do not know
- nausea	Yes	No	Do not know

1c. Have you in connection to recent stomach symptoms had the below

- fever, above 38 degrees	Yes	No	Do not know
- headache	Yes	No	Do not know
- joint pain	Yes	No	Do not know
- pain in eyes	Yes	No	Do not know
- fatigue	Yes	No	Do not know

2. If you have had any of the symptoms in question 1b, state the approximate date for the first onset of disease. Month: Day:

3. Have you fallen ill more than once after you felt recovered for more than 2 days?

No

Yes, at 1 additional occasion

Yes, at 2 additional occasions

Yes, at 3 or more additional occasions

4a. How many days have you in total had stomach symptoms (according to question 1a)?
(Please feel free to use a calender or similar) __ (number of days)

4b. Are you ill at present? Yes No

5. Did you seek health care for the present gastrointestinal disease?

Yes, primary care

Yes, hospital

No

6. Did you call for medical advice regarding the present gastrointestinal disease?

No

Yes, health advice line

Yes, primary care

Yes, hospital

7a. How many days have you been on the sick-list due to gastrointestinal disease since first of January 2011? (Please feel free to use a calender or similar) __ (number of days)

7b. How many days have you been at home due to children with gastrointestinal disease since first of January 2011? (Please feel free to use a calender or similar) __ (number of days)

Section 2: History of disease before first of January 2011

8. Did you have any of the following problems or diseases during 2010:

- gastric ulcer	Yes	No	Do not know
- irritable bowel syndrome (IBS)	Yes	No	Do not know
- inflammatory intestinal disease (Ulcerative colitis or Chron´s disease)	Yes	No	Do not know
- celiac disease	Yes	No	Do not know
- lactose intolerance	Yes	No	Do not know
- other longterm intestinal problems	Yes	No	Do not know
- diabetes	Yes	No	Do not know
- immunodeficiency	Yes	No	Do not know
- chronic obstructive pulmonary disease (COPD)/asthma	Yes	No	Do not know
- congestive heart failure	Yes	No	Do not know
- rheumatic disease	Yes	No	Do not know
- cancer	Yes	No	Do not know

9. Were you treated with any of the following pharmaceuticals during the autumn of 2010:

- medicine for gastric ulcer/acid reflux (e.g. Omeprazol, Losec, Nexium)	Yes	No
- cortison pills	Yes	No
- cytostatics (”cell toxins”) or other pharmaceutical that suppresses the immune system	Yes	No

10. Do you smoke?

Yes, daily

Yes, sometimes

No

Section 3. General questions

11. How many people are there in your household? __ (number of people)

12. How many of these have had gastrointestinal symptoms? (according to question 1a)
__ (number of people)

13. What type of household water supply do you have?*)*

Municipal water

Own well

Other joint facility

Do not know

14. If you have used tap water for drinking or tooth brushing, have you in your household followed the recommendation from the municipality to boil the water since the nineteenth of April?

Yes, always

Yes, sometimes

No

15. When did you start to boil the water? Month: Day:

16. State how many glasses of tap water you usually drink per day on average (do not forget squash, gruel etc. that is made from tap water)? (1 glass is approximately 2 dl)

< 1 glass

1 glass

2-5 glasses

>5 glasses

Do not know

17. Did you answer the web-based questionnaire from the municipality? Yes No

18. A follow-up questionnaire will arrive in September. If we can contact you by e-mail, please state your e-mail address below.

19. Do you want to add anything regarding other problems you have had since first of January 2011 that you think may be due to Cryptosporidium?

In order to remember an incident it may sometimes be easier to obtain some dates that one can relate to. For example I got sick before or after a certain event. Here we give some events and dates that hopefully can help. The first audition to the national song contest in Luleå was on the fifth of February. The first quarter final against Linköping was on the eighth of Mars and Skellefteå AIK went to the final in ice-hockey on the first of April.

Thank you for taking the time to answer the questions!
